# Prevalence of Zinc Deficiency in Japanese Patients on Peritoneal Dialysis: Comparative Study in Patients on Hemodialysis

**DOI:** 10.3390/nu12030764

**Published:** 2020-03-14

**Authors:** Satoshi Shimizu, Ritsukou Tei, Masahiro Okamura, Nobuteru Takao, Yoshihiro Nakamura, Hidetaka Oguma, Takashi Maruyama, Hiroyuki Takashima, Masanori Abe

**Affiliations:** Division of Nephrology, Hypertension and Endocrinology, Department of Internal Medicine, Nihon University School of Medicine, 30-1 Oyaguchi Kami-cho, Itabashi-ku, Tokyo 173-8610, Japan; zeejyo@gmail.com (S.S.); haru_li_huang@yahoo.co.jp (R.T.); patrickemboma@yahoo.co.jp (M.O.); mszero6color@yahoo.co.jp (N.T.); nakayoshi86@gmail.com (Y.N.); jam.kumakuma@gmail.com (H.O.); maruyama.takashi@nihon-u.ac.jp (T.M.)

**Keywords:** dialysis, hemodialysis, peritoneal dialysis, trace elements, zinc

## Abstract

Background: It is known that patients on hemodialysis (HD) are prone to developing zinc deficiency due to removal of zinc by HD, inadequate dietary intake, and reduced gastrointestinal zinc absorption. However, the prevalence of zinc deficiency in patients on peritoneal dialysis (PD) has not been well established. Methods: Serum zinc levels were compared between 47 patients on PD and 47 patients on HD matched for age, sex, and duration of dialysis. A serum zinc level < 60 μg/dL was defined as clinical zinc deficiency and a level of 60–80 μg/dL as subclinical zinc deficiency. The prevalence of zinc deficiency and associated clinical factors were determined in both groups. Results: Clinical zinc deficiency was found in 59.6% of the PD group and 70.2% of the HD group (*p* = 0.391). Subclinical zinc deficiency was found in 40.4% of the PD group and 29.8% of the HD group. Age, body mass index, and serum albumin level were identified as independent predictors of zinc deficiency in the PD group by multivariate analysis. Conclusions: A higher prevalence of clinical and subclinical zinc deficiency was found in patients on PD. The rates were comparable between patients on PD and those on HD after adjustment for confounding factors.

## 1. Introduction

Zinc is an essential trace elements known to play a crucial role in cell metabolism, growth, tissue repair, production of neurotransmitters, and inflammation [1,2,3]. Zinc is also an important cofactor for a number of enzymes, transcriptional factors, and cytokines implicated in several aspects of normal immune system function. Zinc has anti-oxidant and anti-inflammatory properties, regulates T-cell function, and has an important role in maintenance of immune function and combatting infection [4]. It has been reported that zinc deficiency is associated with delayed wound healing and impaired immune function [5,6,7]. Therefore, zinc deficiency might contribute to increased risk of infection in patients undergoing hemodialysis (HD) [8,9,10].

Zinc deficiency may be associated with non-specific symptoms or conditions, which are commonly observed in patients on HD, namely, anorexia, dysgeusia, erythropoiesis-stimulating agent (ESA)-resistant anemia, and impaired cognitive function [11,12,13]. It is known that patients on HD are prone to develop zinc deficiency due to removal of zinc by HD, inadequate dietary intake, and reduced gastrointestinal zinc absorption [14,15]. In contrast, there are few reports on serum zinc concentrations in patients who are undergoing peritoneal dialysis (PD) [16,17]. Prompt detection of zinc deficiency and appropriate zinc supplementation may be beneficial in the dialysis population. Therefore, investigation of the prevalence of zinc deficiency in the PD population is needed. The aim of this study was to compare the prevalence of zinc deficiency and its clinical characteristics between patients on PD and those on HD.

## 2. Subjects and Methods

### 2.1. Patients

**Figure 1:**
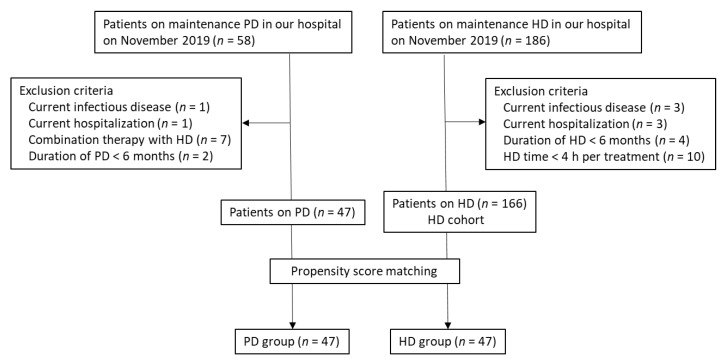
Flowchart showing the selection process for the two study groups. HD, hemodialysis; PD, peritoneal dialysis.

This cross-sectional study analyzed data collected from patients who were on PD or HD at Nihon University Itabashi Hospital from November to December 2019. An overview of the process used to select the study participants is shown in Figure 1. The inclusion criteria were age ≥ 18 years, PD or HD for > 6 months at the time of enrollment, and all medical decisions made at the hospital where the study was performed. The exclusion criteria were as follows: (1) Current hospitalization; (2) apparent malignancy; (3) concurrent infectious disease; (4) liver cirrhosis or chronic hepatitis; (5) concomitant steroid or immunosuppressant medication; (6) gastrointestinal disturbance, such as diarrhea or a malabsorption disorder; history of gastric or bowel resection; (7) concomitant PD and HD; and (8) zinc supplementation within the previous 6 months. Forty-seven patients were enrolled in the PD group after the exclusions. Then, to minimize the confounding factors and compare with PD, the HD group was selected using propensity score matching with adjustments for significant differences in baseline covariates. The HD group was then selected by propensity score matching with adjustment for significant differences in baseline covariates. Propensity scores were calculated for age, sex, duration of dialysis, comorbid cardiovascular disease (CVD), body mass index (BMI), and cause of end-stage kidney disease (ESKD). These scores were used to match patients in the PD group with those in the HD (reference) group in a ratio of 1:1. All patients in the HD group were treated with HD (each session lasting 4–5 h) 3 times weekly. Patients who were found to have received HD < 3 times a week or to have had HD sessions lasting < 4 h per treatment were excluded. After exclusions and propensity score matching, 47 patients were enrolled in the HD group. In addition, serum zinc levels were measured and analyzed for all patients on HD (HD cohort, *n* = 166).

The study protocol was approved by our hospital ethics committee and conducted in accordance with the tenets of the Declaration of Helsinki. All patients provided written informed consent (Clinical Trial Registration No. UMIN000025327).

### 2.2. Data Collection

Demographic and medical data, including age, sex, duration of dialysis, comorbid CVD, and laboratory parameters, were collected from the medical records. Actual body weight (body weight minus PD solution in the body) was measured in the PD group and clinical dry weight was used as the measure of body weight in the HD group. CVD was defined as a history of severe heart failure, coronary artery disease, peripheral artery disease, or stroke. Biochemical parameters, including serum urea nitrogen, serum creatinine, total protein, albumin, electrolytes, total cholesterol, high-density lipoprotein cholesterol, triglycerides, serum iron, total iron binding capacity, intact parathyroid hormone (PTH), and serum ferritin levels, were measured using routine clinical chemistry procedures with commercial kits. C-reactive protein and serum β_2_-microglobulin levels were measured using latex agglutination immunoassay. The serum zinc level was measured by the colorimetric determination method; the normal range was defined as 80–130 μg/dL. The prevalence of zinc deficiency was then investigated. A serum zinc level < 60 μg/dL was defined as clinical zinc deficiency and a level of 60–80 μg/dL as subclinical zinc deficiency according to the Japanese Society of Clinical Nutrition guideline, the results of a nationwide survey, and other literature [18,19,20]. Blood samples were obtained in the HD group before initiation of the first weekly dialysis treatment and in the PD group when patients visited the hospital for routine examinations in the morning. Postprandial blood samples were obtained approximately 1–2 h after a meal in all patients. The same ESA (darbepoetin alfa) was administered in all patients and the monthly dose was recorded. The efficiency of PD and HD was evaluated by Kt/V according to the Japanese Society for Dialysis Therapy guideline (Table S1) [21,22]. Weekly Kt/V urea from residual kidney function (renal Kt/V) and weekly Kt/V urea from PD (PD Kt/V) values were recorded. The sum of renal Kt/V and PD Kt/V was calculated as total Kt/V in patients on PD [21]. Single-pool Kt/V urea (spKt/V) in one dialysis session was calculated for patients on HD [22]. Office blood pressure measurements were obtained in the PD group and before the start of dialysis in the HD group.

### 2.3. Statistical Analysis

Data are expressed as the mean ± standard deviation or as the median (interquartile range) as appropriate. Continuous variables were compared between the study groups using the Student’s *t*-test or Mann–Whitney *U* test and categorical variables using the chi-square test or Fisher’s exact test. Factors associated with the serum zinc level were sought using univariate regression analysis. Sex, age, duration of dialysis, BMI, urine volume, serum urea nitrogen, creatinine, hemoglobin, albumin, sodium, calcium, phosphate, alkaline phosphatase, plasma glucose, C-reactive protein, ferritin, high-density lipoprotein cholesterol, β_2_-microglobulin levels, and Kt/V were selected as independent variables. Multiple logistic regression analysis was then performed using the independent variables that had shown a significant association (*p* < 0.1) with the serum zinc level in the univariate analysis of the PD and HD groups, as well as the entire HD cohort. All statistical analyses were performed using JMP version 12 software (SAS Institute Inc., Cary, NC, USA). A *p*-value < 0.05 was considered statistically significant.

## 3. Results

Table 1 shows the characteristics of the participants in the PD and HD groups. There was no significant difference in age, sex distribution, duration of dialysis, cause of ESKD, or medications between the two groups. There were 14 and 22 anuric patients in the PD and HD groups, respectively (*p* = 0.089). The details of the PD modalities used are shown in Table 2.

Table 3 shows a comparison of laboratory data between the PD and HD groups. Serum total protein, albumin, sodium, and potassium levels were significantly lower in the PD group than in the HD group, whereas total cholesterol and high-density lipoprotein cholesterol levels were significantly higher in the PD group than in the HD group. There was no significant difference in the hemoglobin, transferrin saturation, or β_2_-microglobulin level between the two groups.

**Figure 2:**
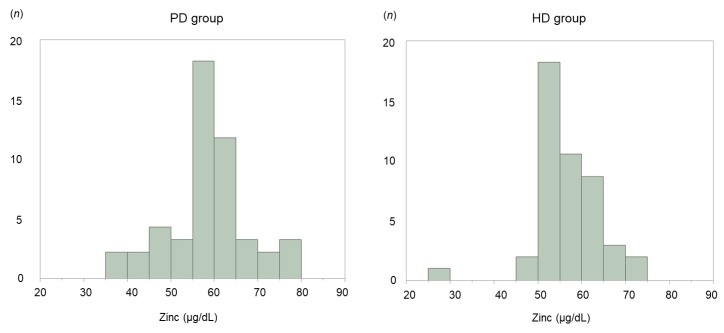
Histogram showing serum zinc concentrations in patients on peritoneal dialysis (PD) and those on hemodialysis (HD).

Serum zinc concentrations were compared between the study groups. Figure 2 shows the distribution of serum zinc levels. There was no significant difference in the mean serum zinc level between the PD and HD groups (58.0 (55.0–63.0) μg/dL vs. 56.0 (53.0–61.0) μg/dL; *p* = 0.346). Figure 2 compares the prevalence of clinical zinc deficiency and subclinical zinc deficiency between the groups. No patient in either group had a normal serum zinc level. There was no significant difference in prevalence of clinical zinc deficiency between the PD and HD groups (59.6% vs. 70.2%; *p* = 0.391) or in the prevalence of subclinical zinc deficiency (40.4% vs. 29.8%; *p* = 0.279). The prevalence of clinical zinc deficiency and subclinical zinc deficiency was comparable between the two groups. In the HD cohort (*n* = 166), mean serum zinc level was 61 ± 10.5 μg/dL, and the prevalence of clinical and subclinical zinc deficiency was 46.4% and 47.6%, respectively. Only 6.0% of the patients had normal serum zinc levels (Figure S1).

Multivariate analysis revealed that age, BMI, and albumin level were independent predictors of serum zinc level in patients on PD (Table 4). In the HD cohort (*n* = 166), univariate analyses revealed that age, BMI, urine volume, creatinine, hemoglobin, albumin, calcium, and spKt/V were associated with serum zinc levels. A multivariate analysis identified that age, BMI, and serum albumin level to be independent predictors of the serum zinc level in all HD patients (Table S2). In the matched HD group, multivariate analysis identified duration of dialysis, BMI, and spKt/V to be independent predictors of serum zinc levels in patients on HD (Table 5).

## 4. Discussion

In this study, the prevalence of clinical zinc deficiency in patients on chronic stable PD was 59.6% and that of subclinical zinc deficiency was 40.4%. Although the prevalence rates of clinical and subclinical zinc deficiency were high in patients on PD, the rate was comparable between the two groups. Furthermore, older age, lower BMI, and a lower serum albumin levels were independent predictors of zinc deficiency in patients on PD. This is the first report to compare the prevalence of zinc deficiency and clinical characteristics between patients on HD and appropriately matched patients on HD.

Approximately 80% of the total zinc in the human body is distributed in erythrocytes and 20% in serum; 60%–80% of serum zinc is bound to albumin [23,24]. In patients with nephrotic syndrome, zinc bound to protein in serum is lost in the urine. Therefore, it was suggested that serum zinc levels were associated with albuminuria in patients with nephrotic syndrome and chronic kidney disease [25]. Alternatively, the rate at which zinc binds to amino acids may increase with decreasing serum albumin levels, and serum zinc levels might also decrease because amino acids that bind zinc are excreted in urine. Therefore, serum zinc concentrations are lower in diabetes patients with nephropathy and proteinuria than in diabetes patients without proteinuria [26,27]. Moreover, it was found that a lower serum zinc level was associated with urinary excretion of albumin-bound zinc in 30 patients on HD [28]. Therefore, it was suggested that a lower serum albumin level was correlated with a lower serum zinc level in patients on HD. However, there was no significant association between serum zinc level and urine volume, which might be explained by the fact that our patients on PD and HD with residual kidney function did not have marked proteinuria. Further investigation is needed to confirm this because we did not measure urinary protein excretion in this study. It has also been reported that the amount of albumin lost during PD is greater than that lost during conventional HD [29,30]. Therefore, serum albumin level was a significant predictor of serum zinc level in patients on PD but not in those on HD.

Patients on PD are as likely to have zinc deficiency as those on HD because of lower serum albumin and peritoneal loss of protein and zinc. Two studies have found that zinc is lost in PD effluent [16,31]. However, in those studies, there was no significant difference in the amount of zinc lost between patients with lower serum zinc levels and those with normal serum zinc levels. Moreover, another study that measured zinc levels in spent PD effluent found no significant peritoneal zinc loss [32]. A different study found that, although the serum zinc level was slightly decreased, the zinc level in erythrocytes was higher in patients on CAPD than in control subjects with normal kidney function [33]. However, no significant difference in the distribution of zinc levels was found in patients with stage four or five chronic kidney disease who were not dependent on dialysis [16,33]. Those reports concluded that PD did not alter the zinc distribution in erythrocytes or plasma. It was also suggested that zinc deficiency might be caused by decreased absorption of zinc from the intestinal tract and poor dietary intake [34,35]. Zinc deficiency in patients on PD might be related to a lower zinc intake because protein restriction is commonly prescribed for patients with ESKD.

A study in 1009 healthy Japanese subjects found that serum zinc levels decreased with age [36]. Specifically, it found that 38% of elderly subjects (aged ≥ 60 years) had a low serum zinc level and that serum zinc level had a positive correlation with zinc intake in the elderly. Furthermore, a U-shaped association has been found between BMI and mortality in younger patients on HD [37]. In contrast, the relationship was not U-shaped in a Japanese study of patients on dialysis, most (74.2%) of whom had a BMI of <23 [38]. A cutoff BMI of 20 is recommended for diagnosis of protein energy wasting in Japanese patients on HD [38], suggesting that lower BMI is associated with mortality, especially in elderly patients on dialysis. It has also been reported that zinc supplementation might improve appetite, stimulate food intake, and increase BMI in patients on HD and that the serum leptin level is decreased in the HD population [39,40,41]. Furthermore, zinc supplementation was shown to increase the total cholesterol level and rate of protein catabolism in patients on HD [42,43]. This is an important issue given the progressive aging of Japanese patients on dialysis. In our study, zinc deficiency was more common in patients who were elderly and those with a low BMI. Further research is needed to determine whether or not zinc supplementation improves malnutrition and other outcomes in elderly patients on dialysis.

Zinc deficiency during periods of growth results in growth failure and the skeletal system is among the organs most affected. Zinc deficiency has been associated with a lower alkaline phosphate level [44] and with short stature and abnormal development [45]. In the present study, alkaline phosphate levels and intact PTH levels were higher in the HD group than in the PD group. Although there were no patients with short stature or abnormal development in our study, it is possible that many patients with secondary hyperparathyroidism were included in the PD group.

This study has several limitations. First, it had a cross-sectional design and included only the small number of patients with PD at a single institution. Therefore, we selected the PD group first and then selected propensity score-matched patients on HD. Larger studies are needed in the future to compare serum zinc levels between patients on PD and those on HD. Second, we could not determine the total amounts of zinc lost via PD effluent, clearance by HD, and loss in urine; therefore, more comparisons of loss of zinc from serum between patients on PD and those on HD are needed. Although BMI was an independent predictor in both study groups, a longer duration of dialysis and a lower Kt/V were associated with a lower serum zinc level only in patients on HD, suggesting a close relationship between the HD procedure and zinc deficiency. Further investigation is needed to clarify whether or not there is a difference in the mechanism of serum zinc loss between PD and HD. Third, blood samples were obtained in all subjects after the morning meal. However, serum zinc levels show diurnal fluctuation, and so it is recommended that the blood samples be obtained early in the morning while in a fasting state. Serum zinc level might be affected by the interval between breakfast and sampling. Finally, given the observational nature of the study, we could not evaluate the efficacy of zinc supplementation. The serum zinc level was identified to be an independent predictor of future hospitalization for infection and of overall mortality in patients on long-term dialysis [46]. More interventional studies are required to confirm the importance of zinc supplementation in patients on PD with zinc deficiency.

## 5. Conclusions

In this study, serum zinc level was similar between PD and HD, and the respective prevalence rates of clinical and subclinical zinc deficiency in patients on PD were comparable with those on HD after controlling for baseline confounding factors. Given the increasing number of elderly patients on dialysis and the finding that age is an independent predictor of zinc levels, further investigations of the efficacy of zinc supplementation in zinc-deficient patients on PD are necessary.

## Figures and Tables

**Table 1:** Patient characteristics and medications at baseline in patients on peritoneal dialysis and those on hemodialysis.

Variable	PD Group	HD Group	*p*-Value
Patients, *n* (male %)	47 (72.3)	47 (70.2)	0.819 ^†^
Age, years	61.3 ± 14.6	62.3 ± 13.9	0.724
Duration of dialysis (months)	22 (9–46)	19 (9–45)	0.893
Comorbid CVD, *n* (%)	5 (10.6)	8 (17.0)	0.368 *
Smoking, *n* (%)	3 (6.4)	5 (10.6)	0.457 *
Alcohol use *n* (%)	5 (10.6)	8 (17.0)	0.370 *
Body mass index, kg/m^2^	23.7 ± 4.0	22.4 ± 3.6	0.107
Cause of ESKD, *n* (%)			
Diabetic nephropathy	14 (29.8)	15 (31.9)	0.823 ^†^
Chronic glomerular nephritis	8 (17.0)	12 (25.6)	0.313 ^†^
Hypertension	23 (48.9)	19 (40.4)	0.406 ^†^
Other	2 (4.3)	1 (2.1)	0.557 *
Anuria, *n* (%)	14 (29.8)	22 (46.8)	0.089 ^†^
CAPD, *n* (%)	22 (46.8)		
APD, *n* (%)	25 (53.2)	-	
PD treatment time, h/day	16.9 ± 4.6	-	
PD fluid volume, L/day	8.0 ± 2.8	-	
PD solution bag exchanges,/day	4.3 ± 1.4	-	
Systolic BP, mmHg	144 ± 19	144 ± 13	0.951
Diastolic BP, mmHg	82 ± 10	81 ± 9	0.784
Heart rate, (bpm)	75 ± 11	74 ± 9	0.816
Medications, *n* (%)			
RAS inhibitor	40 (85.1)	39 (83.0)	0.778 ^†^
Statin	22 (46.8)	20 (42.6)	0.678 ^†^
VDRA	33 (70.2)	38 (80.9)	0.230 ^†^
Phosphate binder	41 (87.2)	45 (95.7)	0.139 ^†^
Calcium carbonate	19 (40.4)	18 (38.3)	0.832 ^†^
Lanthanum carbonate	20 (42.5)	23 (48.9)	0.534 ^†^
Sevelamer hydrochloride	1 (2.1)	3 (6.4)	0.306 *
Ferric citrate	11 (23.4)	9 (19.1)	0.614 ^†^
Sucroferric oxyhydroxide	6 (12.8)	8 (17.0)	0.773 ^†^

APD, automated peritoneal dialysis; BP, blood pressure; CAPD, continuous ambulatory peritoneal dialysis; CVD, cardiovascular disease; ESKD, end-stage kidney disease; HD, hemodialysis; PD, peritoneal dialysis; RAS, renin-angiotensin system; VDRA, vitamin D receptor activator. *Data analyzed by Fisher’s exact test. ^†^ Data analyzed by the chi-square test. The other data were analyzed by the Student’s *t*-test.

**Table 2:** Details of dialysis modalities in the peritoneal dialysis group.

Variable	CAPD	APD
*n* (%)	22 (46.8)	25 (53.2)
PD treatment time, h/day	15 (13.5–20)	18 (14–21)
During the night for APD, h/day	-	8 (7–8)
During the day for APD, h/day	-	10 (6.5–12.8)
PD fluid volume, L/day	6.4 ± 1.7	9.3 ± 2.9
PD solution bag exchanges/day	3.5 (3–4)	5 (4–6)
1.5% glucose dialysate, L/day	3 (2–4.6)	5.6 ± 3.4
2.5% glucose dialysate, L/day	1.5 (0–2)	2 (0–4)
Icodextrin, L/day	2 (1.5–2)	2 (1.5–2)

APD, automated peritoneal dialysis; CAPD, continuous ambulatory peritoneal dialysis; PD, peritoneal dialysis. “-“ means “Patients with CAPD do not use APD during the night and day times” or “N/A”.

**Table 3:** Clinical and laboratory parameters in patients on peritoneal dialysis and those on hemodialysis.

Variable	PD Group	HD Group	*P*-Value
Serum urea nitrogen, mg/dL	54 ± 15	56 ± 12	0.432
Creatinine, mg/dL	10.2 ± 3.8	9.9 ± 3.0	0.630
Total protein, g/dL	6.3 ± 0.7	6.6 ± 0.5	0.012
Albumin, g/dL	3.3 ± 0.5	3.6 ± 0.5	0.008
Sodium, mEq/L	137 ± 3.5	140 ± 2.8	<0.0001
Potassium, mEq/L	4.3 ± 0.6	4.8 ± 0.6	0.0003
Corrected calcium, mg/dL	9.1 ± 0.5	9.4 ± 0.7	0.057
Phosphate, mg/dL	5.6 ± 1.4	5.2 ± 1.2	0.106
Alkaline phosphatase, U/L	266 (217–394)	232 (191–320)	0.020
Intact PTH, pg/mL	230 (119–282)	162 (83–207)	0.009
Total cholesterol, mg/dL	177 ± 39	156 ± 32	0.004
HDL cholesterol, mg/dL	49 ± 36	38 ± 15	0.035
Triglycerides, mg/dL	94 (71–138)	98 (73–155)	0.549
Postprandial plasma glucose, mg/dL	138 ± 30	140 ± 28	0.689
C-reactive protein, mg/dL	0.11 (0.1–0.6)	0.17 (0.05–0.54)	0.378
Hemoglobin, g/dL	11.0 ± 1.2	10.7 ± 0.8	0.195
Iron, μg/dL	82 ± 34	82 ± 30	0.979
Transferrin saturation (%)	33 ± 14	35 ± 14	0.519
Ferritin, ng/mL	112 (37–174)	82 (50–147)	0.898
ESA, μg/m	120 (75–180)	100 (80–160)	0.354
β_2_-microglobulin, mg/L	26 ± 11	24 ± 7	0.260
Renal Kt/V	0.13 (0.05–0.91)	-	-
PD Kt/V	1.13 ± 0.4	-	-
Total Kt/V	1.67 ± 0.7	-	-
spKt/V	-	1.33 ± 0.2	-

ESA, erythropoiesis-stimulating agent; HD, hemodialysis; HDL, high-density lipoprotein; PD, peritoneal dialysis; PTH, parathyroid hormone.

**Table 4:** Univariate and multivariate regression analysis of predictors of the serum zinc level in patients on peritoneal dialysis.

Variable	Univariate			Multivariate				
	Estimate	SE	*p*-Value	Estimate	SE	95% CI		*p*-Value
Age	−0.36	0.07	<0.0001	−0.19	0.06	−0.335	−0.063	0.005
Female sex	0.20	2.97	0.945					
Duration of dialysis	−0.01	0.05	0.862					
Body mass index	1.48	0.22	<0.0001	0.84	0.23	0.364	1.331	0.001
Urine volume	1.56	1.63	0.341					
Serum urea nitrogen	0.02	0.08	0.815					
Creatinine	0.06	0.34	0.841					
Hemoglobin	−0.001	1.08	0.998					
Albumin	4.78	1.63	0.004	4.69	1.79	1.065	8.326	0.012
Sodium	−0.12	0.38	0.750					
Calcium	−2.10	2.52	0.409					
Phosphate	0.13	0.94	0.888					
Alkaline phosphatase	−0.01	0.01	0.241					
Plasma glucose	−0.06	0.04	0.713					
C-reactive protein	−1.19	4.27	0.781					
Ferritin	0.01	0.01	0.348					
HDL-cholesterol	0.09	0.08	0.266					
β_2_-microglobulin	−0.22	0.11	0.055	−0.15	0.07	−0.304	5.890	0.050
Total Kt/V	1.14	1.97	0.565					

CI, confidence interval; HDL, high-density lipoprotein; SE, standard error.

**Table 5:** Univariate and multivariate regression analyses of predictors of the serum zinc level in patients on hemodialysis.

Variable	Univariate			Multivariate				
	Estimate	SE	*p*-Value	Estimate	SE	95% CI		*p*-Value
Age	−0.19	0.07	0.010	−0.02	0.05	−0.138	0.102	0.760
Female sex	4.44	2.31	0.060					
Duration of dialysis	−0.16	0.03	<0.0001	−0.09	0.03	−0.167	−0.031	0.005
Body mass index	1.20	0.21	<0.0001	0.79	0.22	0.346	1.239	0.0009
Urine volume	−0.88	2.81	0.754					
Serum urea nitrogen	0.01	0.09	0.928					
Creatinine	−0.25	0.36	0.484					
Hemoglobin	−1.46	1.26	0.253					
Albumin	7.59	2.02	0.0005	3.52	1.88	−0.282	7.336	0.068
Sodium	−0.35	0.38	0.360					
Calcium	0.13	1.41	0.922					
Phosphate	−0.53	0.93	0.571					
Alkaline phosphatase	−0.001	0.01	0.935					
Plasma glucose	0.02	0.04	0.684					
C-reactive protein	−3.17	2.04	0.127					
Ferritin	−0.001	0.01	0.913					
HDL-cholesterol	−0.07	0.06	0.291					
β_2_-microglobulin	−0.18	0.14	0.187					
spKt/V	10.8	5.32	0.047	9.59	3.70	2.118	17.06	0.013

CI, confidence interval; HDL, high-density lipoprotein; SE, standard error.

## Supplementary Materials

The following are available online at https://www.mdpi.com/2072-6643/12/3/764/s1, Figure S1: Serum zinc concentrations in the HD cohort (*n* = 166), Table S1: Methods used to evaluate the dose of peritoneal dialysis and hemodialysis, Table S2: Univariate and multivariate regression analysis of predictors of serum zinc level in the HD cohort (*n* = 166).
