# Supplementary material for: Prevalence of Zinc Deficiency in Japanese Patients on Peritoneal Dialysis: Comparative Study in Patients on Hemodialysis
Source: Nutrients. 2020 Mar 14;12(3):764. doi: 10.3390/nu12030764 (PMC7146559; doi:10.3390/nu12030764)
Supplement: Supplementary file 1 [file nutrients-12-00764-s001.zip › Table S1.docx]

**Table S1.** Methods used to evaluate the dose of peritoneal dialysis and hemodialysis.

**Methods used to evaluate the peritoneal dialysis (PD) dose**

| ***Urea Kt/V* (*Kt/Vurea*)** |
| --- |
| 1. The usual dialysis fluid is injected. |
| 2. After *t* (min), the fluid is drained as usual, the drainage volume (*V_D_*(*t*)) is recorded, and a sample of drained dialysis fluid (*C_D_*(*t*)) is collected. |
| 3. [1] and [2] above are repeated for the day. |
| 4. Blood is sampled on the same day and the blood urea nitrogen concentration (*C_B_*) is determined. |
| 5. The amount of urea nitrogen (*V_D_*(*t*) × *C_D_*(*t*)) eliminated for the day (four times) is summed and the total is divided by *C_B_*. |
| 6. The *Kt/V* for the day is calculated by dividing the result of [5] by the body fluid volume (*V_B_*). *V_B_* (mL) is calculated from the correlations with height (*HT* [cm]) and body weight (*BW* [kg]). The following are typical correlation equations.  Hume and Weyers equation ^1^:  *V_B_* = 194.786 × *HT* + 296.785 × *BW* − 14012.934 (male patients)  *V_B_* = 344.547 × *HT* + 183.809 × *B*W − 35270.121 (female patients)  Watson and Watson equation ^2^:  *V_B_* = 107.4 × *HT* + 336.2 × *BW* + 2447 − 95.16 × age (male patients)  *V_B_* = 106.9 × *HT* + 246.6 × *BW* − 2097 (female patients) |
| 7. The *Kt/V* for the week is calculated by multiplying the result of [6] by 7. |

**Methods used to evaluate the hemodialysis (HD) dose**

| **Single-pool Kt/Vurea (spKt /V)** |
| --- |
| Kt/Vurea is an index of the degree to which urea is removed in one hemodialysis session. Daugirdas proposed several definitive equations for Kt/Vurea^3^. The following equation assumes the one-compartment model with consideration of the effect of fluid removal and production of urea.  spKt/V = −ln (R − 0.008t) + (4 − 3.5R) ×ΔV/BWpost  where R is the ratio of the postdialysis sUN to the predialysis sUN concentration (= sUNpost/sUNpre), t is the dialysis duration (h), ΔV is the fluid removal per hemodialysis session (L), and BWpost is the patient’s postdialysis BW (kg). |

**References**

1. Hume R, Weyers E. Relationship between total body water and surface area in normal and obese subjects. J Clin Path 1971; 24: 234–8.

2. Watson PE, Watson ID, Batt RD. Total body water volumes for adult males and females estimated from simple anthropometric measurements. Am J Clin Nutr 1980; 33: 27–39.

3. Daugirdas JT. The post: pre-dialysis plasma urea nitrogen ratio to estimate Kt/V and NPCR: mathematical modeling. Int J Artif Organs 1989; 12: 411–9.
