# Supplementary material for: Prevalence of Zinc Deficiency in Japanese Patients on Peritoneal Dialysis: Comparative Study in Patients on Hemodialysis
Source: Nutrients. 2020 Mar 14;12(3):764. doi: 10.3390/nu12030764 (PMC7146559; doi:10.3390/nu12030764)
Supplement: Supplementary file 1 [file nutrients-12-00764-s001.zip › Table S2.docx]

**Table S2.** Univariate and multivariate regression analysis of predictors of serum zinc level in the HD cohort (*n* = 166).

| Variable | Univariate | | |  | Multivariate | | | | |
| --- | --- | --- | --- | --- | --- | --- | --- | --- | --- |
|  | Estimate | SE | P-value |  | Estimate | SE | 95% CI | | P-value |
| Age | −0.25 | 0.05 | < 0.0001 |  | −0.18 | 0.06 | −0.31 | −0.06 | 0.0026 |
| Female sex | 1.02 | 1.60 | 0.522 |  |  |  |  |  |  |
| Duration of dialysis | 0.01 | 0.01 | 0.151 |  |  |  |  |  |  |
| Body mass index | 0.64 | 0.20 | 0.002 |  | 0.62 | 0.20 | 0.22 | 1.02 | 0.0025 |
| Urine volume | 4.14 | 2.46 | 0.093 |  | 4.35 | 2.25 | −0.09 | 8.81 | 0.055 |
| Serum urea nitrogen | 0.05 | 0.05 | 0.264 |  |  |  |  |  |  |
| Creatinine | 0.61 | 0.25 | 0.017 |  | −0.11 | 0.29 | −0.69 | 0.46 | 0.692 |
| Hemoglobin | 2.11 | 0.78 | 0.007 |  | 1.14 | 0.84 | −0.54 | 2.82 | 0.180 |
| Albumin | 12.5 | 2.53 | < 0.0001 |  | 11.0 | 2.79 | 5.51 | 1.6.5 | 0.001 |
| Sodium | −0.04 | 0.23 | 0.838 |  |  |  |  |  |  |
| Calcium | 2.42 | 1.32 | 0.070 |  | 1.96 | 1.27 | −0.55 | 4.47 | 0.125 |
| Phosphate | 0.54 | 0.63 | 0.389 |  |  |  |  |  |  |
| Alkaline phosphatase | −0.001 | 0.01 | 0.912 |  |  |  |  |  |  |
| Plasma glucose | 0.01 | 0.02 | 0.798 |  |  |  |  |  |  |
| C-reactive protein | 0.74 | 1.91 | 0.696 |  |  |  |  |  |  |
| Ferritin | 0.01 | 0.01 | 0.214 |  |  |  |  |  |  |
| HDL-cholesterol | 0.09 | 0.08 | 0.266 |  |  |  |  |  |  |
| β_2_-microglobulin | 0.05 | 0.11 | 0.625 |  |  |  |  |  |  |
| spKt/V | 3.81 | 1.84 | 0.040 |  | 2.29 | 1.88 | −1.50 | 5.96 | 0.239 |

HDL, high density lipoprotein
