# Supplementary figures and images for: Prevalence of Zinc Deficiency in Japanese Patients on Peritoneal Dialysis: Comparative Study in Patients on Hemodialysis
Source: Nutrients. 2020 Mar 14;12(3):764. doi: 10.3390/nu12030764 (PMC7146559; doi:10.3390/nu12030764)

**Figure S1.** Serum zinc concentrations in the HD cohort (*n* = 166)


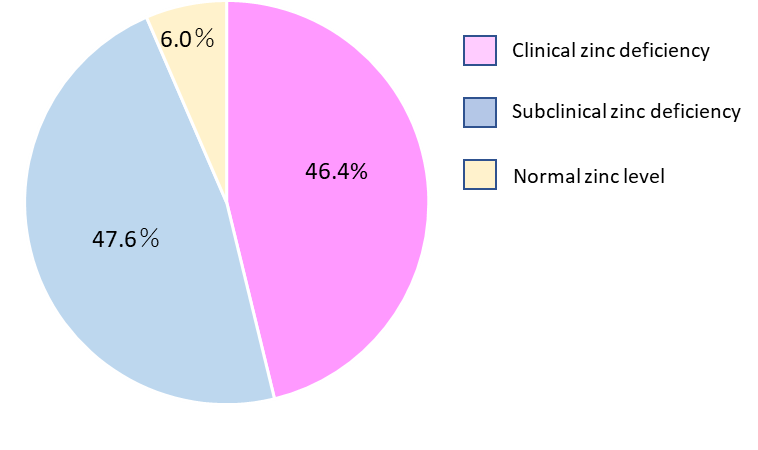

Supplement: Supplementary file 1 [file nutrients-12-00764-s001.zip › Figure S1.docx]
